# Widely metastatic *IDH1*-mutant glioblastoma with oligodendroglial features and atypical molecular findings: a case report and review of current challenges in molecular diagnostics

**DOI:** 10.1186/s13000-019-0793-5

**Published:** 2019-02-09

**Authors:** Carlos G. Romo, Doreen N. Palsgrove, Ananyaa Sivakumar, Christen R. Elledge, Lawrence R. Kleinberg, Kaisorn L. Chaichana, Christopher D. Gocke, Fausto J. Rodriguez, Matthias Holdhoff

**Affiliations:** 1 Brain Cancer Program, Sidney Kimmel Comprehensive Cancer Center at Johns Hopkins, 1550 Orleans Street, 1M16, Baltimore, MD 21287 USA; 2 Department of Pathology, Johns Hopkins University of Medicine, Baltimore, MD USA; 3 Department of Radiation Oncology, Johns Hopkins University of Medicine, Baltimore, MD USA; 4 Department of Neurosurgery, Johns Hopkins University of Medicine, Baltimore, MD USA

**Keywords:** High grade glioma, 1p/19q-codeletion, Whole arm deletion, *IDH1*, *ATRX*, Tumor lysis syndrome, Metastatic, MYC

## Abstract

**Background:**

Gliomas with 1p/19q-codeletion as well as mutation of *isocitrate dehydrogenase* (*IDH*) *1* are typically characterized as oligodendrogliomas with comparatively good response to treatment with radiation and chemotherapy.

**Case presentation:**

We present the case of a 28-year-old man with an *IDH1* and *TP53* mutant high grade glioma with abnormalities in chromosomes 1 and 19 suggestive of anaplastic oligodendroglioma that rapidly progressed to widespread metastatic disease. Biopsy of a liver lesion confirmed metastasis of the patient’s known brain primary and chemotherapy with temozolomide was initiated. The patient’s rapidly growing tumor burden with fulminant liver failure and tumor lysis led to multisystem failure of which the patient died. Further molecular testing illustrated features more consistent with glioblastoma: multiple large chromosomal aberrations including loss of whole chromosome 1 and 2q; gain/amplification of *MYCN*, *MET*, and *CDK4*; loss of *CDKN2A/B*; and an *ATRX* mutation.

**Conclusion:**

This case illustrates the importance of higher level molecular diagnostic testing for patients with particularly aggressive disease progression that is not concordant with standard prognoses. Additional data on cases with atypical alterations of 1p and 19q are needed to better understand the distinct biology of these cancers so that appropriate therapies can be developed.

**Electronic supplementary material:**

The online version of this article (10.1186/s13000-019-0793-5) contains supplementary material, which is available to authorized users.

## Background

The understanding of molecular features of diffuse gliomas has been revolutionized by the discovery of two distinct molecular markers: complete deletion of both the short arm of chromosome 1 (1p) and the long arm of chromosome 19 (19q) (1p/19q-codeletion) in oligodendroglial tumors, as well as mutations of the *isocitrate dehydrogenase* (*IDH*) *1* or *2* genes in oligodendrogliomas and a subset of astrocytic tumors [1–3]. The clinical relevance of these molecular features in anaplastic oligodendrogliomas was determined in two randomized clinical trials, the Radiation Therapy Oncology Group (RTOG) 9402 and the European Organization for Research and Treatment of Cancer (EORTC) study 26,951, with evidence of 1p/19q-co-deletion as determined by fluorescence in-situ hybridization (FISH) analysis. Both studies showed a significant survival benefit when chemotherapy with lomustine, procarbazine, and vincristine (PCV) was added to radiation [1, 4], whereas there has not been a significant benefit for those without 1p/19q-codeletion. Further analysis of the data illustrated that patients whose tumors harbored an *IDH1* mutation in addition to a 1p/19q-codeletion fared better overall compared to patients with co-deletion alone [5]. This molecular alteration is the result of an unbalanced whole-arm translocation between chromosomes 1 and 19 with the loss of the derivative t (1p;19q) early in the pathogenesis of oligodendrogliomas. Although the biological effect of 1p/19q-codeletion remains unclear, it is considered the diagnostic molecular signature of oligodendrogliomas.

There are several different methods that have been used to identify patients whose tumors harbor 1p/19q-codeletion; however, there is no consensus over which test should be used clinically. Although FISH is the preferred method, there is variation in the chromosomal loci targeted and the definitions and cut-offs used to determine chromosome deletion. FISH is also unable to differentiate between whole chromosome arm deletions and smaller focal deletions. Array comparative genomic hybridization (aCGH) and single nucleotide polymorphism (SNP) array are capable of identifying loss of the whole arm of 1p or 19q with higher reliability but introduce additional interpretative challenges in samples with low tumor purity or high tumor heterogeneity [6].

Here we present a case of a young man with a high grade *IDH1*-mutant glioma with abnormalities in chromosomes 1 and 19 who experienced an unusually aggressive disease progression following standard therapy for anaplastic oligodendrogliomas. This case illustrates the clinical impact that variations of otherwise favorable prognostic markers can have on clinical outcome and it highlights current molecular diagnostic challenges in characterizing glial neoplasms.

## Case presentation

**Fig. 1:**
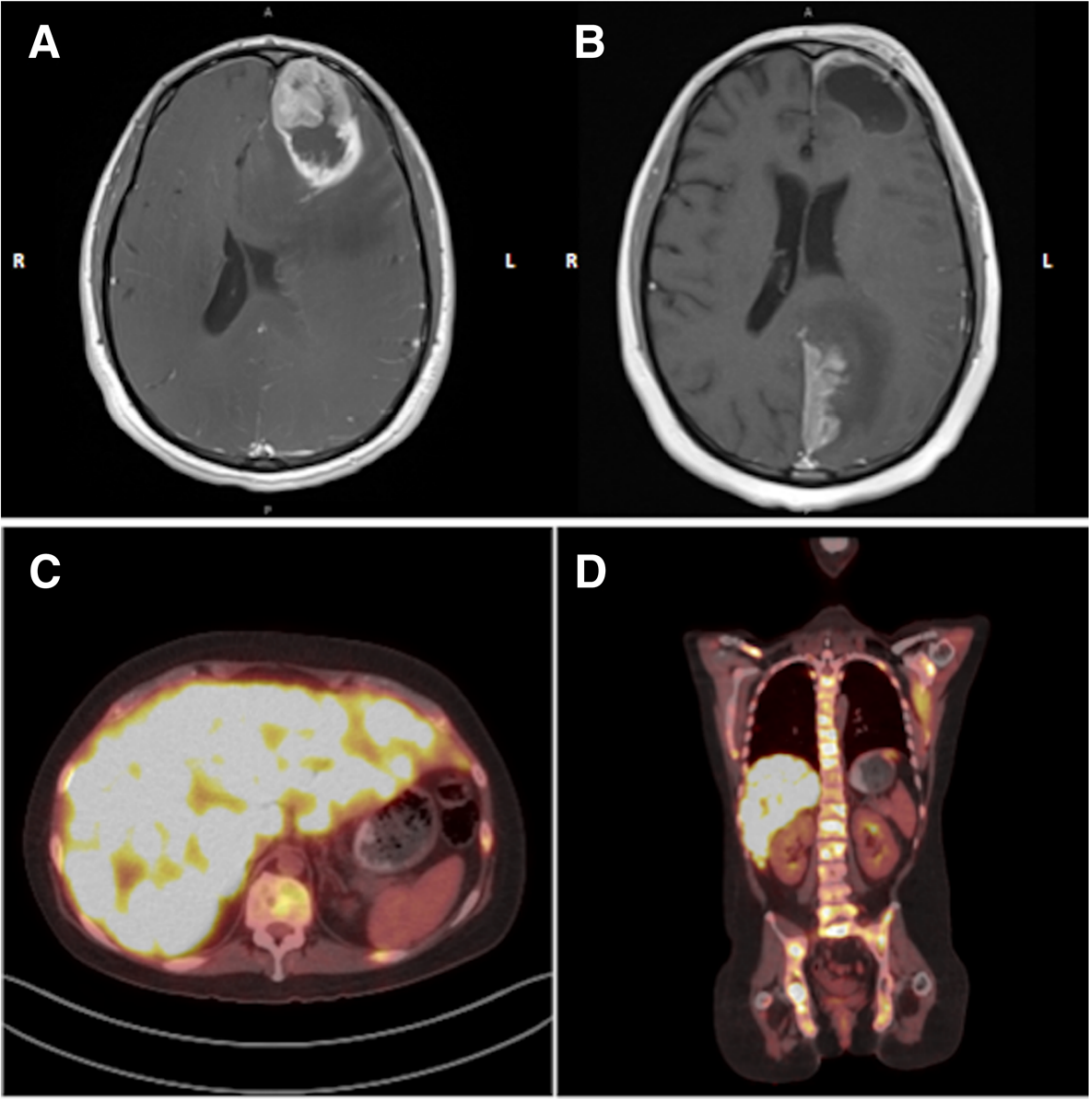
Axial MRI brain post administration of gadolinium **a** on initial presentation with a large contrast enhancing lesion and a necrotic core in the left frontal lobe with mass effect. **b** Images 2 weeks after completion of radiation therapy showing an enhancing lesion involving the paramedian left parietal-occipital lobe extending to the superior surface of the left cerebellar tentorium. **c** axial and **d** coronal FDG PET scan with hypermetabolic lytic bony lesions throughout the axial and proximal appendicular skeleton as well as innumerable hyperdensities in the liver concerning for metastatic disease

**Fig. 2:**
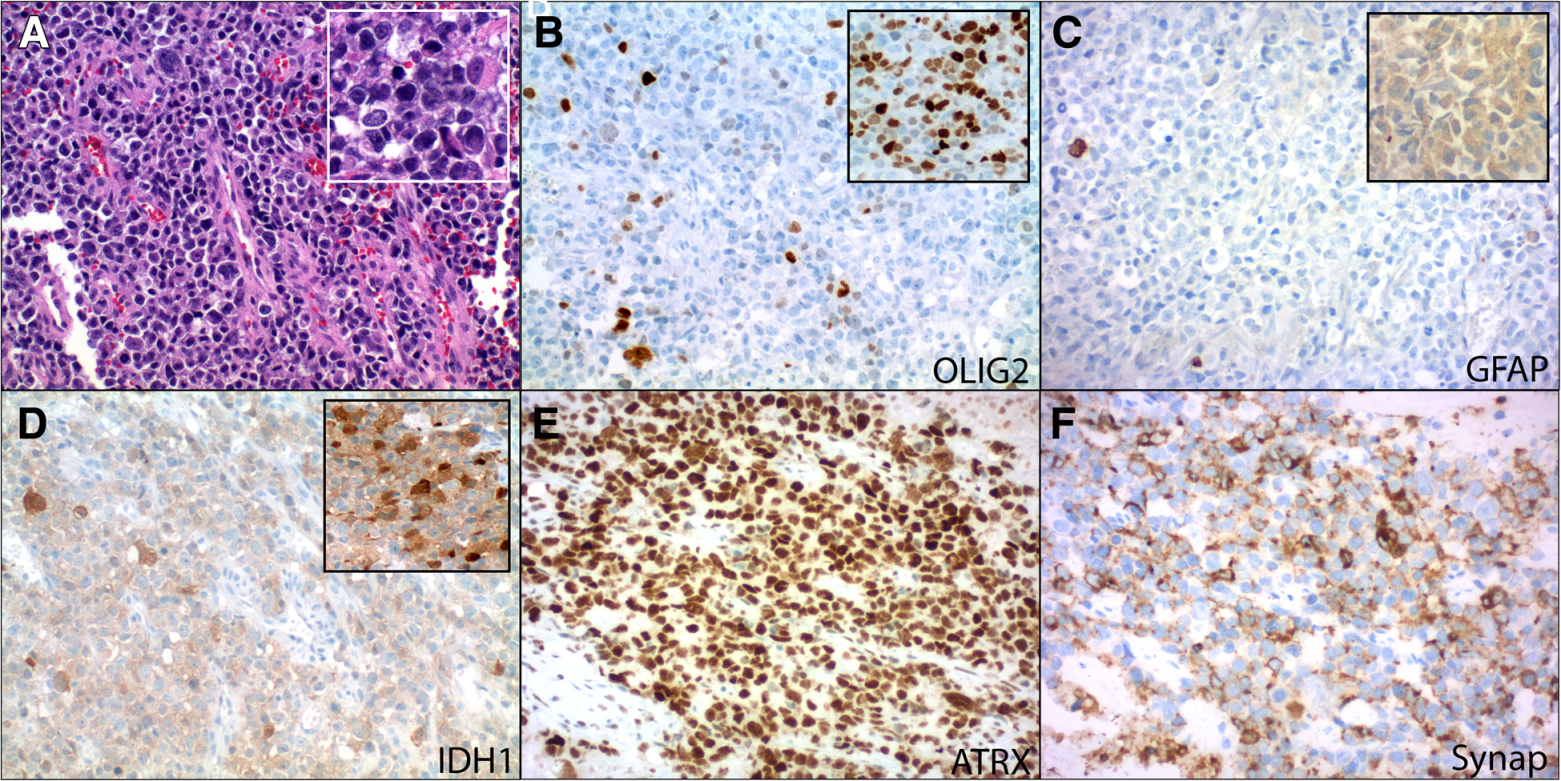
Routine hematoxylin and eosin stains (H&E) showed infiltrative sheets of discohesive neoplastic cells (**a**, 200X; inset, 400X). The majority of cells demonstrated high nuclear to cytoplasmic ratios with round hyperchromatic nuclei and moderate nuclear pleomorphism. A subset of cells showed gemistocytic morphology with eccentrically displaced dense eosinophilic cytoplasm (**a**, 200X; inset, 400X). Occasional giant cells were also noted. Ancillary immunohistochemical stains showed that the neoplasm contained some areas of IDH1-mutant protein positive cells and other areas that were predominantly IDH1-mutant protein negative to weakly positive (**d**, 200X; inset, 200X). GFAP (**c**, 200X; inset, 200X) and OLIG2 (**b**, 200X; inset, 200X) expression was also variable. ATRX protein expression was uniformly retained throughout (**e**, 200X) and synaptophysin was diffusely positive (**f**, 200X). The neoplasm also co-expressed NeuN and S-100 protein, while chromogranin was focal (not shown). INI-1 and BRG1 were retained. Additional stains for CD99, HH3 K27 M, SOX10, CD30, myogenin, desmin, CAM5.2 (CK8/18), AE1/AE3, OCT3/4, Melan A, CD45, CD3, and CD20 were negative in the tumor

**Fig. 3:**
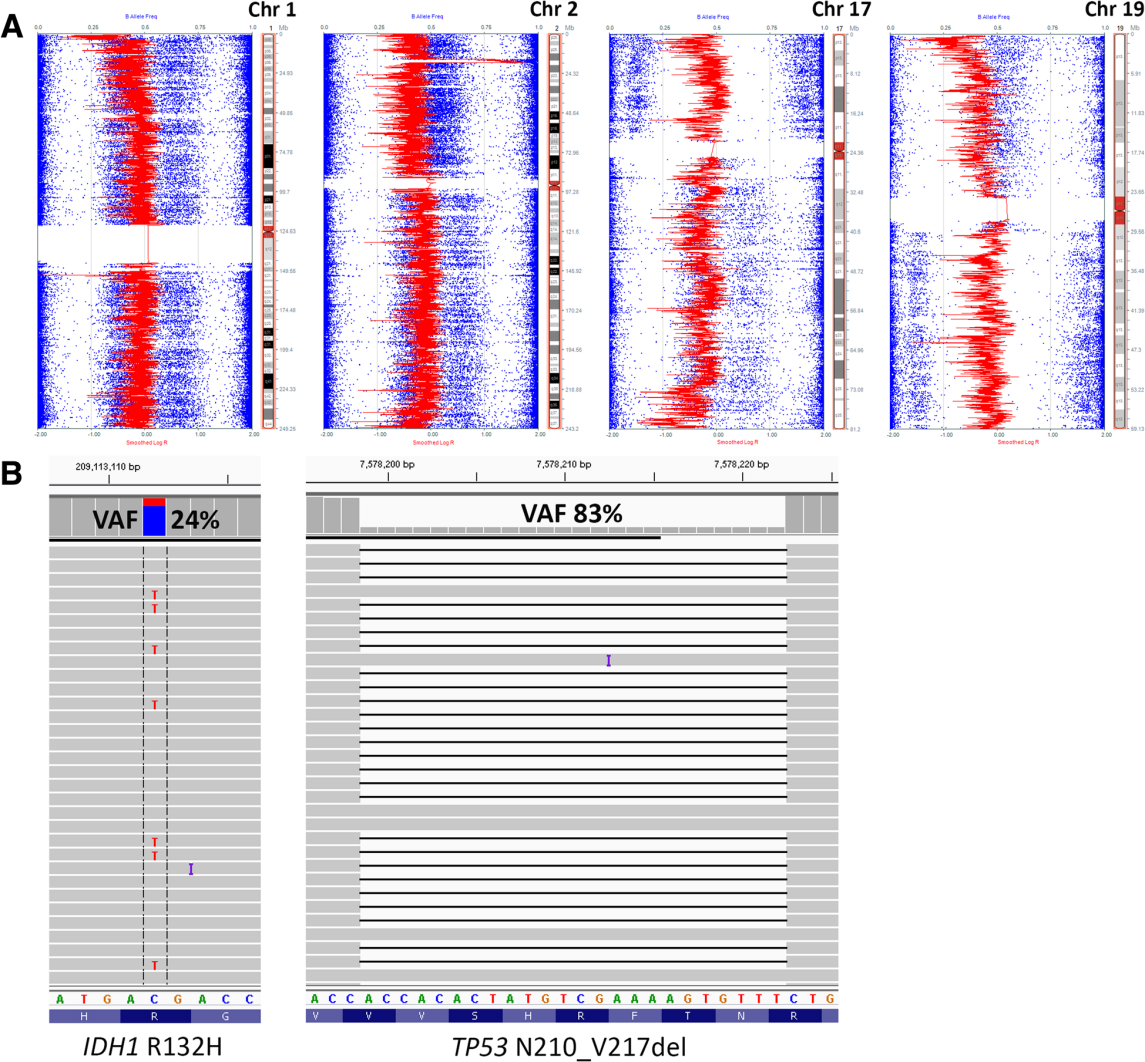
Single nucleotide polymorphism (SNP) array data using Illumina HumanCytoSNP-850 K (v1.1) BeadChip platform (approximately 850,000 SNPs) and iScan microarray system and illustrated with KaryoStudio v2.0 software; red data show smoothed signal intensity values (LRR) (Log base 2 ratio of observed and expected intensities; LogR 0, copy number two) and blue data points represent the B-allele frequency (BAF) of each individual SNP (B-allele frequency of 0 equals no B-allele; 1 equals only B-alleles present). Loss of chromosomal segments is supported by the downward shift of the red vertical line (decrease in LRR, left shift) and loss of heterozygosity (LOH) in BAF (loss of heterozygous BAF track around 0.5 with variable redistribution of BAF in in region of LOH associated with the ratio of tumor to normal DNA in the sample), while gains/amplifications of genomic regions show upward shifts of the red vertical line (increase in LRR, right shift) and LOH in BAF. Loss of whole chromosome 1, 2q, 17p, and the majority of 17q and 19q is depicted here (**a**) as well as gain/amplification of 2p24.2-p24.3, which includes *MYCN*. Initial amplicon-based targeted next generation sequencing using the Ion AmpliSeq Cancer Hotspot Panel v2 demonstrated an *IDH1* hotspot mutation (p.R132H) and an in-frame *TP53* deletion (p.N210_V217del), visualized here using Integrated Genomics Viewer v2.3.4 (IGV; Broad Institute, MIT Harvard) (**b**). Subsequent SNP array analysis demonstrated loss of whole arm 17p (*TP53* is located at 17p13.1) in the vast majority of the sample; therefore, the *TP53* VAF can be used to approximate tumor purity. The *IDH1* mutation, on the other hand, appears to be present in only a fraction of tumor cells, while *IDH1* wild type cells comprise the remainder of the tumor with apparent loss of the mutant *IDH1* allele (*IDH1* is located at 2q34). Matched normal (tissue or peripheral blood) was not available for comparison

The patient was a 28-year-old man who presented with a 2-week history of subtle personality changes, expressive aphasia, and severe headaches with nausea and vomiting. Imaging revealed a large mass in the left frontal lobe with significant surrounding vasogenic edema (Fig. 1a). He underwent gross total resection and pathology was consistent with a high grade neuroepithelial tumor with brisk mitotic activity and necrosis (Fig. 2, Additional file 1 : Figures S1 and S2). The tumor was found to be *IDH1* and *TP53* mutant by amplicon-based next generation sequencing (NGS) targeting the hotspot regions of 27 cancer associated genes (not including *ATRX*), and had monosomy 1 and 19q loss by single nucleotide polymorphism (SNP) array (Figs. 2 and 3). A diagnosis of anaplastic oligodendroglioma was favored based on histologic appearance of the majority of the tissue with features suggestive of an oligodendroglioma in addition to preserved ATRX immunoreactivity, and atypical alterations in chromosomes 1 and 19. Treatment options were discussed including participation in the CODEL trial (NCT00887146), but ultimately started standard of care treatment with radiation therapy with plan for adjuvant procarbazine, lomustine, and vincristine (PCV) chemotherapy following radiation. Unfortunately, after resection and prior to starting radiation he developed somnolence, an intense headache, and diplopia. MRI of the brain revealed evidence of tumor regrowth in the original location and signs of impending herniation. He was started on dexamethasone and underwent emergent surgical decompression with tumor debulking. Pathology was again consistent with a high-grade *IDH1-*mutant glioma. His symptoms improved and he received intensity-modulated radiation therapy (IMRT) with a cumulative dose of 5940 cGy over 33 fractions. MGMT promoter methylation was tested upon recurrence and was found to be present.

Approximately two weeks after completing IMRT he experienced two focal seizures with secondary generalization resulting in right hemiparesis. Repeat MRI of the brain showed evidence of disease progression with interval development of a new enhancing lesion outside the radiation field involving the paramedian left parietal-occipital lobe extending to the superior surface of the left cerebellar tentorium (Fig. 1b). Due to newly reported low back pain, he underwent MRI of the spine which demonstrated extensive bony metastases. A whole-body ^18^Fluorodeoxyglucose (FDG) PET-CT scan was obtained and revealed innumerable hypermetabolic lesions throughout the patient’s body including lytic lesions virtually involving his entire skeleton and innumerable lesions in the liver, right lung, and left kidney. His clinical condition rapidly deteriorated and developed a clinical picture consistent with tumor lysis syndrome. Lactate dehydrogenase was elevated on admission at 5206 U/L (normal range 118–273 U/L) and increased to a peak level of 15,080 U/L. He became hyperuricemic (peak of 12.7 mg/dL; normal range 3.5–7.2 mg/dL) and hypophosphatemic (1.1 mg/dL; normal range 2.7–4.5 mg/dL) with evidence of acute kidney injury (creatinine from 0.6 mg/dL on admission to a peak of 1.9 mg/dL). Rasburicase was administered to treat his hyperuricemia. A biopsy of one of the liver masses in the right hepatic lobe (3.6 cm) was performed to rule out the presence of an independent neoplastic process given the very unusual aggressiveness of the cancer and widespread metastatic disease. The tissue sample showed prominent cellular necrosis with rare clusters of neoplastic cells, IDH1-mutant protein positive, consistent with metastasis of the patient’s known brain primary.

In addition to tumor lysis, the patient rapidly became thrombocytopenic (179 K/cu mm on admission to a nadir of 35 K/cu mm by day seven) and anemic (14.5 g/dL to a nadir of 5.8 g/dL), likely due to bone marrow infiltration of his cancer. Different chemotherapy regimens were considered and the choice was for temozolomide, as it is typically effective in diffuse gliomas and is able to cross the blood-brain barrier with even higher concentrations systemically. Five doses were planned (150 mg/m^2^) but the patient was only able to receive two administrations.

Unfortunately, the patient’s condition continued to deteriorate. He developed worsening renal failure, hypotension, and acute respiratory failure. He was intubated and mechanically ventilated, requiring vasopressor support. The patient developed cardiac arrest, was resuscitated and eventually transitioned to comfort measures. He was terminally extubated and passed away. An autopsy was declined.

Further analysis of the patient’s tumor tissue using a targeted, capture-based next generation sequencing panel covering the full coding regions of 644 cancer associated genes showed the presence of a number of additional genetic alterations (Table 1), including an *ATRX* missense mutation, and amplification of *MYCN*, *MET*, and *CDK4*. There were no mutations in *CIC* or *FUBP1*. Other chromosomal variations, including gain of 7p and loss of 2q, 10p, 11p, 21q, and whole chromosomes 12 and 14 were also noted (Additional file 1: Table and Figure S3).

**Table 1:**
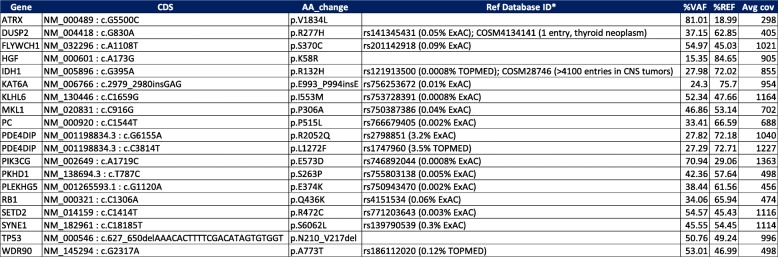
Hybrid Capture-Based Targeted Next Generation Sequencing of 644 Cancer Associated Genes (full coding regions)

*CDS* coding DNA sequence, *AA change* amino acid change, *VAF* variant allele frequency, *REF* reference allele frequency, *Avg cov* average depth of coverage across all gene-specific exonic targets. Some of the variants with an allele frequency around 50% may be germline; however, the high tumor cellularity (> 90%) of the specimen precludes the distinction of germline from somatic alterations based on %VAF alone

*dnSNP v150 and COSMIC database v82

## Discussion and conclusions

The development of extracranial metastasis from primary gliomas is rare (up to 2.7% at one institution) [7–12] and typically occurs late in the course of the disease after a median of 2 years. The average patient age associated with glioblastoma-related metastasis has been demonstrated to be around 40 years [13]. The most common metastatic sites include the pleura/lung (60%), lymph nodes (51%), bone (31%), and liver (22%). Concerning bone metastasis, Pasquier et al., reported that the vertebral spine (73%) was the most frequent site of involvement, followed by the ribs (23%) and sternum (18%) [14]. To date, clinicopathological factors that would predict a greater likelihood of metastasis have not been identified; however, there appears to be no significant difference in median survival between GBM patients with and without metastatic disease [3]. Few molecular analyses of metastatic lesions have been previously attempted [3, 15, 16], but all have been limited in scope. Further studies with larger sample sizes are needed to elucidate the molecular mechanisms underlying metastatic potential. The identification of such biomarkers may prompt earlier screening for systemic disease in high grade glioma patients.

Despite being *IDH1*-mutated and *MGMT* promoter methylated, both of which have been correlated with favorable outcome in malignant gliomas, this was an unusually aggressive clinical course for an IDH-mutated tumor with whole arm 1p and 19q abnormalities. For patients with prototypical 1p/19q-codeleted IDH-mutant tumors, overall survival has been shown to be significantly prolonged by combined PCV and radiation therapy compared to radiation therapy alone (14.7 versus 6.8 years, HR 0.49, 95% CI 0.28–0.85) [17, 18]. Temozolomide is also viewed as a reasonable option for 1p/19q-codeleted tumors. It has better patient tolerance compared to the short-term toxicity of PCV and has been shown to improve survival in other types of malignant gliomas. Additionally, both PCV and temozolomide have demonstrated activity in patients who have failed an initial chemotherapy regimen although response rates are lower and the duration of disease control is generally shorter compared to treatment at first diagnosis [19]. *MGMT* promoter methylation is also independently predictive of therapeutic benefit from combined PCV and radiation with a significantly longer median overall survival (8.65 years) compared to radiation therapy alone (1.98 years) in patients with *MGMT* methylated tumors [20].

Review of extended molecular analyses from this case illustrates several atypical features that exemplify some of the ongoing diagnostic challenges that arise when attempting to integrate molecular testing results into clinical treatment paradigms. Foremost, the chromosome 1 and 19 alterations are not typical of those seen in oligodendrogliomas. Instead of a deletion involving only the short arm of chromosome 1, both the short and long arms were deleted in a subset of cells, indicating the presence of monosomy 1. 1p/19q-codeletion represents an unbalanced centromeric whole-arm chromosomal translocation (with the loss of the derivative chromosome) t (1;19) (q10;p10) in which both the entire 1p and 19q arm are lost. The lack of equivalent allelic imbalances in 1p and 19q in this case favors the absence of a 1p and 19q chromosomal translocation. Vogazianou et al. followed strict guidelines when determining patterns of copy number alterations involving chromosomes 1 and 19. 1p/19q-codeletion was only called in the presence of concurrent total 1p loss and total 19q loss and pericentromeric regions of 1q and 19p showed no signs of deletion (e.g. monosomy 1 or 19 were not included). Following these rules, monosomy 1 was detected in only 2 of the 363 astrocytic and oligodendroglial tumors analyzed and were categorized as either anaplastic astrocytoma or glioblastoma [21]. Other notable chromosomal alterations in this case include gain of 7p (without high level amplification of *EGFR*) and del(9p21), which are frequently encountered in glioblastomas [6]. Molecular reclassification of this case as a secondary glioblastoma was considered. Rare fragments of adjacent *IDH1*-mutated lower grade glioma (Additional file 1: Figure S1, and Additional file 1: Figure S2a) were identified in the resection specimens. Furthermore, the presence of concurrent IDH and *TP53* mutations are now considered molecular hallmark features of diffuse and anaplastic astrocytomas (WHO grades II and III), as well as clinicopathologically defined secondary glioblastoma [22].

Also characteristic of diffuse adult IDH-mutant astrocytic tumors are truncating *ATRX* mutations with loss of nuclear ATRX expression by immunohistochemistry. In this case, however, ATRX immunoexpression was retained and the *ATRX* mutation was not definitively deleterious, despite being located in an evolutionarily highly conserved functional domain of the gene (Snf2 ATPase) and classified as probably damaging/disease causing by three in silico prediction callers (PolyPhen-2, MutationTaster, SIFT/PROVEAN). A closer a look at the literature reveals that somatic *ATRX* missense mutations are detected in a subset of adult gliomas [23–25]. Interpretation of ATRX staining is especially problematic in these cases, since tumors with missense mutations that result in loss of protein function will continue to show protein expression and nuclear localization. Similarly, not all insertions/deletions or nonsense will show loss of nuclear ATRX expression [25]. Further complicating diagnostic interpretation are the lack of standard criteria for what constitutes loss of ATRX expression, which is often used as a surrogate marker for *ATRX* mutations in gliomas, and the presence of intratumoral heterogeneity [26].

Some histologic and molecular features in this case were reminiscent of glioblastoma with primitive neuronal component, although not entirely typical. Glioblastoma with primitive neuronal component is a newly recognized pattern in the WHO 2016 classification that includes otherwise classical high-grade diffuse gliomas with one or more foci of sharply demarcated primitive nodules showing neuronal differentiation. Similar to the primitive neuronal cells that make up CNS embryonal neoplasms, these foci show immunoreactivity for synaptophysin, loss of GFAP expression, and a high Ki-67 proliferation index, which were also noted in our case. Two particularly distinctive features of this pattern are its high rate of CSF dissemination and frequent *MYCN* or *MYC* gene amplification (17 of 40 cases, 43%), often in the setting of histologic anaplasia, as seen in medulloblastomas with large cell/anaplastic features [22, 27, 28]. A subset of cases may also demonstrate features of secondary glioblastoma, including IDH1(R132H)-mutant protein expression. The vast majority of the tumor in the present case was composed of sheets of cells with rounded cytoplasmic contours without a pattern, variable amounts of eosinophilic cytoplasm with eccentrically-placed large nuclei, and variably prominent nucleoli (Additional file 1: Figure S2). Areas with nodular architecture and a relatively sharp transitional interface between low- and high-grade glial components were also noted in one fragment (Additional file 1: Figure S2A). Another entity on the histologic differential that can be excluded based on available molecular data is epithelioid glioblastoma (IDH-wildtype, and *BRAF* V600E mutant in 50% of cases, with a subset of *BRAF* V600E negative cases are characterized by *PDGFRA* amplification) [29]. Previous editions of the WHO classification of tumors of the central nervous system included the diagnosis of oligoastrocytoma; its use is now discouraged unless molecular testing cannot be performed or when results are inconclusive in a neoplasm composed of two distinct cell types resembling astrocytic and oligodendroglial cell lines and was therefore considered but not used in this case [2].

Despite improved overall outcomes compared to IDH-wildtype gliomas, many WHO grade II-III *IDH1*-mutant gliomas eventually progress to more aggressive GBMs. Small cohort studies, however, have provided some insight into the mechanisms behind *IDH1*-mutant glioma progression [30–33]. Recently, Bai et al. compared mutation, copy number, gene expression, and DNA methylation data of 41 *IDH1*-mutant grade II and III gliomas (including oligodendrogliomas and astrocytomas) obtained at their initial diagnosis to their progressed counterparts and found that amplification of the *MYC* locus (22% of patients) on chromosome 8q was significantly associated with progression as were alterations in the individual MYC signaling components FBXW7 and MAX and the MYC-related *FOXM1* locus on 12p. Of note, deletion of the *CDKN2A*-*CDKN2B* tumor suppressor locus was the most frequent copy number alteration detected in the cohort, occurring in 44% of all tumors analyzed [30]. Neither amplification of *MYC* nor deletion of *CDKN2A-CDKN2B* were shown to be enriched in either of the oligodendroglial or astrocytic subtypes. Another recent study of paired primary and recurrent IDH-mutant gliomas reported allelic imbalances of the IDH-mutant allele upon recurrence in some cases [31]. Cells with *IDH1* copy number alterations (deletion or amplification of the mutant or wild-type allele) were also shown to occupy a substantial portion of the recurrent tumor in these cases, suggesting a growth advantage, as well as have hypomethylation of non-CpG island CpG sites. A similar case reported by Favero et al. details malignant progression of an *IDH1*-mutant glioma through whole-genome doubling, evolution of a double-minute chromosome containing *PDGFRA*, *KIT*, *CDK4*, *AVIL*, and miR-26a-2 (regulator of *PTEN*), and loss of the mutated *IDH1* allele with retention of the wild-type allele [34]. Our case also shows subclonal loss of the *IDH1*-mutant allele, suggesting that acquisition of additional driver events, such as *MYCN* amplification, have occurred to uphold the tumor cell population [35, 36].

The extensive subclonality of chromosomal aberrations in this case, as evidenced by the variable LOH shifts in B allele frequencies on SNP array (Additional file 1: Figure S3), is not surprising given current data on intratumoral genotypic and phenotypic heterogeneity in glioblastomas [36–39]. Not only are copy number alterations variably present, but some putative driver aberrations, such as gain/amplification of *PDGFRA*, are also known to be consistently heterogenous within the same tumor [39]. Furthermore, there is accumulating evidence suggesting that intratumoral heterogeneity, including branched tumor evolution involving genetically distinct subclones and/or multiple cancer stem cell populations, is the main confounding factor behind tumor recurrence, progression, and treatment failure by current modalities [30, 31, 34, 36]. Of particular interest are non-lineage specific alterations implicated as drivers of higher grade and progressive IDH-mutant diffuse gliomas and associated with shorter progression-free survival [32]. Regardless of whether these transformative alterations are present in the initial tumor sampled or subsequently discovered in the progressive tumor specimen, their presence is important to recognize as it provides an opportunity for the use of novel targeted therapies and combinations thereof [33, 36].

Amplification of *MYCN*, as seen in this case, has been previously reported in a subset of glioblastomas and IDH-mutant gliomas by multiple groups [27, 33, 39–45]. Upon retrospective univariate analysis of 211 IDH-mutated astrocytomas, Shirahata et al. found *MYCN* amplification (*n* = 12, *P* = 0.001) and homozygous deletion of *CDKN2A/B* (*n* = 38, *P* = 0.0001) to be strongly associated with worse overall survival [44]. In contrast, analysis by Perry et al. of malignant gliomas with primitive neuronal components (MG-PNET), which are enriched for *MYCN* amplifications, showed no difference in overall survival between MG-PNETs and conventional glioblastoma [27]. However, their analysis did not stratify cases by the presence or absence of *MYCN* amplification. Amplification of *MYCN* is also associated with poor prognosis in medulloblastomas [4, 46], and strongly predicts a poorer prognosis in both time to tumor progression and overall survival in all stages of neuroblastoma [47, 48]. High grade diffuse midline gliomas, H3-K27 M mutant, are also particularly enriched for *MYCN*, *MET*, and *CDK4* amplifications and usually characterized by poor clinical outcome [49–52].

Our own analysis of the TCGA Merged Cohort of LGG and GBM dataset [45, 53] revealed *MYCN* amplification in 18 of 794 (2%) sequenced cases/patients with copy number data. The average age at diagnosis was 51 years (median 48 years, range 25 to 75 years). Of those with available survival data (*n* = 14), 6 were deceased with a median survival of 30 months (versus 34.9 months among those without *MYCN* amplification). *IDH1* mutations were present in 39% (7 of 18) of *MYCN* amplified glioma cases and *TP53* mutations in 67% (12 of 18). Concurrent amplifications were also present in *EGFR* (*n* = 5, all IDH-wildtype) and *PDGFRA* (n = 5, two of which also had *EGFR* amplification). All *MYCN* amplified cases were 1p/19q-non-codeleted and showed no gain of chromosome 19 or 20. Additional analysis of the GENIE Cohort v4.0 (http://www.cbioportal.org/genie, last accessed Aug 7, 2018, The AACR Project GENIE Consortium), similarly demonstrated *MYCN* amplifications in 1.59% (33 of 2079) of profiled patients classified as glioblastoma, glioblastoma multiforme, anaplastic astrocytoma, astrocytoma, oligodendroglioma, anaplastic oligodendroglioma, high-grade glioma NOS, low-grade glioma NOS, diffuse glioma, anaplastic oligoastrocytoma, oligoastrocytoma, gliosarcoma, small cell glioblastoma*,* glioma NOS, or diffuse astrocytoma [54]. *IDH1/2* mutations were present in 45% (15 of 33) of cases. The most frequent co-amplification involved *EGFR* in 27% of cases (9 of 33, two of which also had concurrent *IDH1* mutations), followed by *PDGFRA* in 24% (8 of 33, two of which also had *EGFR* amplification). Excluding two pediatric cases (< 18 years), the average age at which sequencing was reported was 51 years (median 54 years, range 24 to 78 years).

Unfortunately, the development of inhibitors targeting MYC/MYCN proteins, which are composed of two extended alpha-helices with no obvious surfaces for small molecular binding, has been challenging. Alternative strategies that circumvent blocking MYCN directly include: blocking MYCN-dependent transcription with BET-bromodomain inhibitors; inhibiting histone deacetylases and EZH2 (which repress transcription of tumor suppressor genes); antagonizing proteins involved in stabilizing MYCN protein (e.G. *aurora* kinase A); suppressing MDM2 (which stabilizes *MYCN* mRNA and disrupts p53-mediated apoptosis); and inducing differentiation as a means to suppress proliferation and promote apoptosis [55, 56]. These strategies, although promising in preclinical models, are still under evaluation in ongoing clinical trials.

This case highlights some of the existing challenges in neuro-oncology and the importance of molecular diagnostic interpretation standards for diffuse gliomas. First, although there is growing knowledge regarding the importance of 1p/19q-codeletion and *IDH1* mutations in the clinical care of patients with oligodendrogliomas, we still have limited understanding of how to address cases with less common aberrations involving discordant clonal populations with 1p and/or 19q deletion and deletions involving both arms of either chromosome 1 or 19. Second, the diagnostic use of immunohistochemical surrogates for lineage specific molecular genetic alterations in gliomas is not as straight forward as one might think, especially considering the lack of strict phenotype-genotype relationships for a variety of underlying genetic alterations. Therefore, standard criteria are also needed for immunohistochemical interpretation of diagnostic markers given the potential impact “mis-interpretation” may have on therapeutic decision-making and outcome assessment. Lastly, current molecular testing guidelines for gliomas by the National Comprehensive Cancer Network® (NCCN®) are limited to 1p/19q-codeletion or loss of heterozygosity (LOH), *IDH1/2* mutations, and *MGMT* promoter methylation [57]. This case highlights that there may be benefits to additional extended molecular testing beyond these standard validated molecular biomarkers, particularly for patients with high risk features, unresectable disease, or tumor recurrence despite standard-of-care treatment. While the NCCN encourages any cancer patient to participate in clinical trials, these trials are often based on the presence or absence of specific genetic targets for which clinical testing has not been uniformly established. Potentially informative genetic markers, such as *MYCN* amplification, have not yet gained prognostic relevance in clinical trials, likely due to their relatively infrequent occurrence and/or lack of effective pharmacologic actionability.

As deep molecular genetic characterization becomes more widely available, it is important to take stock of clinically and molecularly challenging cases. Making their data available in the literature and dedicated databases will hopefully lead to further research and the development of more personalized therapies for patients with very rare, molecularly unique tumors.

## Additional file

Additional file 1: **Figure S1.** Routine hematoxylin and eosin stains (H&E) show separate and adjacent areas of lower grade (WHO grades II to III) infiltrating glioma (A, 200X; B, 200X). These areas were positive for IDH1 (R132H) mutant protein by ancillary immunohistochemical staining (C). **Figure S2.** Hematoxylin and eosin stains with neoplastic tissue largely composed of patternless sheets of cells (A, 100X; B, 40X; C, 200X) with rounded cytoplasmic contours, variable amounts of eosinophilic cytoplasm with minimal to no stellate cellular processes (D, 400X; E, 400X; F, 200X), large nuclei, and variably prominent nucleoli (D). Necrosis was predominantly zonal and frequently associated with sclerosed or thrombosed blood vessels (B, 40X). Rare foci suspicious for vascular invasion were also noted (E, 400X). Rare fragments showed adjacent areas of lower grade, infiltrating glioma (A). **Figure S3.** Single nucleotide polymorphism (SNP) array data using Illumina HumanCytoSNP-850K (v1.1) BeadChip platform (approximately 850,000 SNPs) and iScan microarray system and illustrated with KaryoStudio v2.0 software; red data show smoothed signal intensity values (LRR) (Log base 2 ratio of observed and expected intensities; LogR 0, copy number two) and blue data points represent the B-allele frequency (BAF) of each individual SNP (B-allele frequency of 0 equals no B-allele; 1 equals only B-alleles present). Loss of chromosomal segments is supported by the downward shift of the red vertical line (decrease in LRR, left shift) and loss of heterozygosity (LOH) in BAF (loss of heterozygous BAF track around 0.5 with variable redistribution of BAF in in region of LOH associated with the ratio of tumor to normal DNA in the sample), while gains/amplifications of genomic regions show upward shifts of the red vertical line (increase in LRR, right shift) and LOH in BAF. Other chromosomal variations, including gain of 7p and loss of 2q, 10p, 11p, 21q, and whole chromosomes 12 and 14 were noted. **Table S1.** Next generation sequencing panel covering the full coding regions of 644 cancer associated genes showed the presence of a number of additional genetic alterations. (DOCX 3476 kb)

## Abbreviations

aCGH: Array comparative genomic hybridization
CNS: Central nervous system
CSF: Cerebrospinal fluid
EGFR: Epidermal growth factor receptor
EORTC: European Organization for the Research and Treatment of Cancer
FISH: Florescence in situ hybridization
GBM: Glioblastoma multiforme
GENIE: Genomics Evidence Neoplasia Information Exchange
IDH: Isocitrate dehydrogenase
IMRT: Intensity-modulated radiation therapy
LGG: low-grade glioma
LOH: Loss of heterozygosity
MGMT: Methylguanine methyltransferase
MG-PNET: Malignant glioma-primitive neuroectodermal tumor
MRI: Magnetic resonance imaging
NCCN: National Comprehensive Cancer Network
NOS: Not otherwise specified
PCR: Polymerase chain reaction
PCV: Procarbazine, lomustine (also known as CCNU®) vincristine
PDGFRA: Platelet-derived growth factor receptor alpha
SNP: Single nucleotide polymorphism
TCGA: The Cancer Genome Atlas
WHO: World Health Organization

## References

[1] Cairncross JG, Ueki K, Zlatescu MC, Lisle DK, Finkelstein DM, Hammond RR (1998). Specific genetic predictors of chemotherapeutic response and survival in patients with anaplastic oligodendrogliomas. J Natl Cancer Inst.

[2] Louis DN, Perry A, Reifenberger G, von Deimling A, Figarella-Branger D, Cavenee WK (2016). The 2016 World Health Organization classification of tumors of the central nervous system: a summary. Acta Neuropathol.

[3] Park CC, Hartmann C, Folkerth R, Loeffler JS, Wen PY, Fine HA (2000). Systemic metastasis in glioblastoma may represent the emergence of neoplastic subclones. J Neuropathol Exp Neurol.

[4] Tomlinson FH, Jenkins RB, Scheithauer BW, Keelan PA, Ritland S, Parisi JE (1994). Aggressive medulloblastoma with high-level N-myc amplification. Mayo Clin Proc.

[5] Cairncross G, Jenkins R (2008). Gliomas with 1p/19q codeletion: a.K.A. oligodendroglioma. Cancer J.

[6] Crespo I, Vital AL, Nieto AB, Rebelo O, Tão H, Lopes MC (2011). Detailed characterization of alterations of chromosomes 7, 9, and 10 in glioblastomas as assessed by single-nucleotide polymorphism arrays. J Mol Diagn.

[7] Amitendu S, Mak SK, Ling JM, Ng WH (2012). A single institution experience of the incidence of extracranial metastasis in glioma. J Clin Neurosci.

[8] Demeulenare M, Duerinck J, Du Four S, Fostier K, Michotte A, Neyns B (2016). Bone marrow metastases from a 1p/19q co-deleted oligodendroglioma – A case report. Anticancer Res.

[9] Han SR, Yoon SW, Yee GT, Choi CY, Lee DJ, Sohn MJ (2008). Extraneural metastases of anaplastic oligodendroglioma. J Clin Neurosci.

[10] Jian Y, Gao W, Wu Y, Li Y, Zhang Y, Yang G (2016). Oligodendroglioma metastasis to the bone marrow mimicking multiple myeloma: a case report. Oncol Lett.

[11] Kural C, Pusat S, Sentürk T, Seçer Hİ, Izci Y (2011). Extracranial metastases of anaplastic oligodendroglioma. J Clin Neurosci.

[12] Liwnicz BH, Rubinstein LJ (1979). The pathways of extraneural spread in metastasizing gliomas: a report of three cases and critical review of the literature. Hum Pathol.

[13] Goodwin CR, Liang L, Abu-Bonsrah N, Hdeib A, Elder BD, Kosztowski T (2016). Extraneural Glioblastoma Multiforme Vertebral Metastasis. World Neurosurg.

[14] Pasquier B, Pasquier D, N’Golet A, Pahn MH, Couderc P (1980). Extraneural metastases of astrocytomas and glioblastomas. Clincopathological study of two cases and review of the literature. Cancer.

[15] Anghileri E, Castiglione M, Nunziata R, Boffano C, Nazzi V, Acerbi F (2016). Extraneural metastases in glioblastoma patients: two cases with YKL-4-positive glioblastomas and a meta-analysis of the literature. Neurosurg Rev.

[16] Li G, Zhang Z, Zhang J, Jin T, Liang H, Gong L (2014). Occipital anaplastic oligodendroglioma with multiple organ metastases after a short clinical course: a case report and literature review. Diagn Pathol.

[17] Cairncross G, Wang M, Shaw E, Jenkins R, Brachman D, Buckner J (2013). Phase III trial of chemoradiotherapy for anaplastic oligodendroglioma: long-term results of RTOG 9402. J Clin Oncol.

[18] Holdhoff M, Cairncross GJ, Kollmeyer TM, Zhang M, Zhang P, Mehta MP (2017). Genetic landscape of extreme responders with anaplastic oligodendroglioma. Oncotarget.

[19] Wick W, Roth P, Hartmann C, Hau P, Nakamura M, Stockhammer F (2016). Long-term analysis of the NOA-04 randomized phase III trial of sequential radiochemotherapy of anaplastic glioma with PCV or temozolomide. Neuro-Oncology.

[20] van den Bent MJ, Brandes AA, Taphoorn MJ, Kros JM, Kouwenhoven MC, Delattre JY (2013). Adjuvant procarbazine, lomustine, and vincristine chemotherapy in newly diagnosed anaplastic oligodendroglioma: long-term follow-up of EORTC brain tumor group study 26951. J Clin Oncol.

[21] Vogazianou AP, Chan R, Bäcklund LM, Pearson DM, Liu L, Langford CF (2010). Distinct patterns of 1p and 19q alterations identify subtypes of human gliomas that have different prognoses. Neuro-Oncol.

[22] Louis DN, Ohgaki H, Wiestler OD (2016). WHO Classification of Tumours of the Central Nervous System.

[23] Jiao Y, Killela PJ, Reitman ZJ, Rasheed BA, Heaphy CM, de Wilde RF (2012). Frequent ATRX, CIC, FUBP1 and IDH1 mutations refine the classification of malignant gliomas. Oncotarget.

[24] Liu X, Gerges N, Korshunov A, Sabha N, Khuong-Quang D, Fontebasso AM (2012). Frequent ATRX mutations and loss of expression in adult diffuse astrocytic tumors carrying IDH1/IDH2 and TP53 mutations. Acta Neuropathol.

[25] Yamamichi A, Ohka F, Aoki K, Suzuki H, Kato A, Hirano M (2018). Immunohistochemical ATRX expression is not a surrogate for 1p19q codeletion. Brain Tumor Pathol.

[26] Tanboon J, Williams EA, Louis DN (2016). The diagnostic use of immunohistochemical surrogates for signature molecular genetic alterations in gliomas. J Neuropathol Exp Neurol.

[27] Perry A, Miller CR, Gujrati M, Scheithauer BW, Zambrano SC, Jost SC (2009). Malignant gliomas with primitive neuroectodermal tumor-like components: a clinicopathologic and genetic study of 53 cases. Brain Pathol.

[28] Swartling FJ, Savov V, Persson AI, Chen J, Hackett CS, Northcott PA (2012). Distinct neural stem cell populations give rise to disparate brain tumors in response to N-MYC. Cancer Cell.

[29] Korshunov A, Chavez L, Sharma T, Ryzhova M, Schrimpf D, Stichel D, et al. Epithelioid glioblastomas stratify into established diagnostic subsets upon integrated molecular analysis. Brain Pathol. 2017. 10.1111/bpa.12566. 10.1111/bpa.12566 PMC7469088 28990704

[30] Bai H, Harmancı AS, Erson-Omay EZ, Li J, Coşkun S, Simon M (2016). Integrated genomic characterization of IDH1-mutant glioma malignant progression. Nat Genet.

[31] Mazor T, Chesnelong C, Pankov A, Jalbert LE, Hong C, Hayes J (2017). Clonal expansion and epigenetic reprogramming following deletion or amplification of mutant IDH1. Proc Natl Acad Sci U S A.

[32] Richardson TE, Snuderl M, Serrano J, Karajannis MA, Heguy A, Oliver D, et al. Rapid progression to glioblastoma in a subset of IDH-mutated astrocytomas: a genome-wide analysis. J Neuro-Oncol. 2017. 10.1007/s11060-017-2431-y. 10.1007/s11060-017-2431-y 28421459

[33] Wakimoto H, Tanaka S, Curry WT, Loebel F, Zhao D, Tateishi K (2014). Targetable signaling pathway mutations are associated with malignant phenotype in IDH-mutant gliomas. Clin Cancer Res.

[34] Favero F, McGranahan N, Salm M, Birkbak NJ, Sanborn JZ, Benz SC (2015). Glioblastoma adaptation traced through decline of an IDH1 clonal driver and macro-evolution of a double-minute chromosome. Ann Oncol.

[35] Johannessen TA, Mukherjee J, Viswanath P, Ohba S, Ronen SM (2016). Rapid conversion of mutant IDH1 from driver to passenger in a model of human gliomagenesis. Mol Cancer Res.

[36] McGranahan N, Favero F, de Bruin EC, Birkbak NJ, Szallasi Z, Swanton C. Clonal status of actionable driver events and the timing of mutations processes in cancer evolution. Sci Transl Med. 2015. 10.1126/scitranslmed.aaa1408. 10.1126/scitranslmed.aaa1408 PMC4636056 25877892

[37] Piccirillo SG, Combi R, Cajola L, Patrizi A, Redaelli S, Bentivegna A (2009). Distinct pools of cancer stem-like cells coexist within human glioblastomas and display different tumorigenicity and independent genomic evolution. Oncogene.

[38] Soeda A, Hara A, Kunisada T, Yoshimura S, Iwama T, Park DM (2015). The evidence of glioblastoma heterogeneity. Sci Reports.

[39] Sottoriva A, Spiteri I, Piccirillo SG, Touloumis A, Collins VP, Marioni JC (2013). Intratumor heterogeneity in human glioblastoma reflects cancer evolutionary dynamics. Proc Natl Acad Sci U S A.

[40] Beroukhim R, Getz G, Nghiemphu L, Barretina J, Hsueh T, Linhart D (2007). Assessing the significance of chromosomal aberrations in cancer: methodology and application to glioma. Proc Natl Acad Sci U S A.

[41] Brennan CW, Verhaak RG, McKenna A, Campos B, Noushmehr H, Salama SR (2013). The somatic genomic landscape of glioblastoma. Cell.

[42] Hui AB, Lo KW, Yin XL, Poon WS, Ng HK (2001). Detection of multiple gene amplifications in glioblastoma multiforme using array-based comparative genomic hybridization. Lab Investig.

[43] Sanborn JZ, Salama SR, Grifford M, Brennan CW, Mikkelsen T, Jhanwar S (2013). Double minute chromosomes in glioblastoma multiforme are revealed by precise reconstruction of oncogenic amplicons. Cancer Res.

[44] Shirahata M, Ono T, Stichel D, Schrimpf D, Reuss DE, Sahm F (2018). Novel, improved grading system (s) for IDH-mutant astrocytic gliomas. Acta Neuropathol.

[45] The Cancer Genome Atlas Research Network (2015). Comprehensive, integrative genomic analysis of diffuse lower grade gliomas. N Engl J Med.

[46] Pfister S, Remke M, Benner A, Mendrzyk F, Toedt G, Felsberg J (2009). Outcome prediction in pediatric medulloblastoma based on DNA copy-number aberrations of chromosomes 6q and 17q and the MYC and MYCN loci. J Clin Oncol.

[47] Cohn SL, Pearson AD, London WB, Monclair T, Ambros PF, Brodeur GM (2009). The international neuroblastoma risk group (INRG) classification system: an INRG task force report. J Clin Oncol.

[48] Luksch R, Castellani MR, Collini P, De Bernardi B, Cont M, Gambini C (2016). Neuroblastoma (peripheral neuroblastic tumors). Crit Rev Oncol Hematol.

[49] Buczkowicz P, Hoeman C, Rakopoulos P, Pajovic S, Letourneau L, Dzamba M (2014). Genomic analysis of diffuse intrinsic pontine gliomas identifies three molecular subgroups and recurrent activating ACVR1 mutations. Nat Genet.

[50] Mackay A, Burford A, Carvalho D, Izquierdo E, Fazal-Salom J, Taylor KR (2017). Integrated Molecular Meta-Analysis of 1,000 Pediatric High-Grade and Diffuse Intrinsic Pontine Glioma. Cancer Cell.

[51] Wu G, Diaz AK, Paugh BS, Rankin SL, Ju B, Li Y (2014). The genomic landscape of diffuse intrinsic pontine glioma and pediatric non-brainstem high-grade glioma. Nat Genet.

[52] Pierscianek D, Kim YH, Motomura K, Mittelbronn M, Paulus W, Brokinkel B (2013). MET gain in diffuse astrocytomas is associated with poorer outcome. Brain Pathol.

[53] Ceccarelli M, Barthel FP, Malta TM, Sabedot TS, Salama SR, Murray BA (2016). Molecular profiling reveals biologically discrete subsets and pathways of progression in diffuse glioma. Cell.

[54] The AACR Project GENIE Consortium (2017). AACR project GENIE: powering precision medicine through an international consortium. Cancer Discov.

[55] Huang M, Weiss WA (2013). Neuroblastoma and MYCN. Cold Spring Harbor Perspec Med.

[56] Hutter S, Bolin S, Weishaupt H, Swartling FJ (2017). Modeling and targeting MYC genes in childhood brain tumors. Genes.

[57] Nabors LB, Portnow J, Ammirati M, Baehring J, Brem H, Butowski N (2017). NCCN guidelines insights: central nervous system cancers, version 1.2017. J Natl Compr Cancer Netw.

[58] Ambinder EB, Rowe SP (2017). A Case of Anaplastic Oligodendroglioma With Extensive Extraneural Metastases Imaged With FDG PET. Clin Nucl Med.

