# Supplementary material for: Widely metastatic IDH1-mutant glioblastoma with oligodendroglial features and atypical molecular findings: a case report and review of current challenges in molecular diagnostics
Source: Diagn Pathol. 2019 Feb 9;14:16. doi: 10.1186/s13000-019-0793-5 (PMC6368694; doi:10.1186/s13000-019-0793-5)
Supplement: Supplementary file 1 — Figure S1. Routine hematoxylin and eosin stains (H&E) show separate and adjacent areas of lower grade (WHO grades II to III) infiltrating glioma (A, 200X; B, 200X). These areas were positive for IDH1 (R132H) mutant protein by ancillary immunohistochemical staining (C). Figure S2. Hematoxylin and eosin stains with neoplastic tissue largely composed of patternless sheets of cells (A, 100X; B, 40X; C, 200X) with rounded cytoplasmic contours, variable amounts of eosinophilic cytoplasm with minimal to no stellate cellular processes (D, 400X; E, 400X; F, 200X), large nuclei, and variably prominent nucleoli (D). Necrosis was predominantly zonal and frequently associated with sclerosed or thrombosed blood vessels (B, 40X). Rare foci suspicious for vascular invasion were also noted (E, 400X). Rare fragments showed adjacent areas of lower grade, infiltrating glioma (A). Figure S3. Single nucleotide polymorphism (SNP) array data using Illumina HumanCytoSNP-850K (v1.1) BeadChip platform (approximately 850,000 SNPs) and iScan microarray system and illustrated with KaryoStudio v2.0 software; red data show smoothed signal intensity values (LRR) (Log base 2 ratio of observed and expected intensities; LogR 0, copy number two) and blue data points represent the B-allele frequency (BAF) of each individual SNP (B-allele frequency of 0 equals no B-allele; 1 equals only B-alleles present). Loss of chromosomal segments is supported by the downward shift of the red vertical line (decrease in LRR, left shift) and loss of heterozygosity (LOH) in BAF (loss of heterozygous BAF track around 0.5 with variable redistribution of BAF in in region of LOH associated with the ratio of tumor to normal DNA in the sample), while gains/amplifications of genomic regions show upward shifts of the red vertical line (increase in LRR, right shift) and LOH in BAF. Other chromosomal variations, including gain of 7p and loss of 2q, 10p, 11p, 21q, and whole chromosomes 12 and 14 were noted. Table S1. Next generati [file 13000_2019_793_MOESM1_ESM.docx]

**Supplemental Figure 1**

**
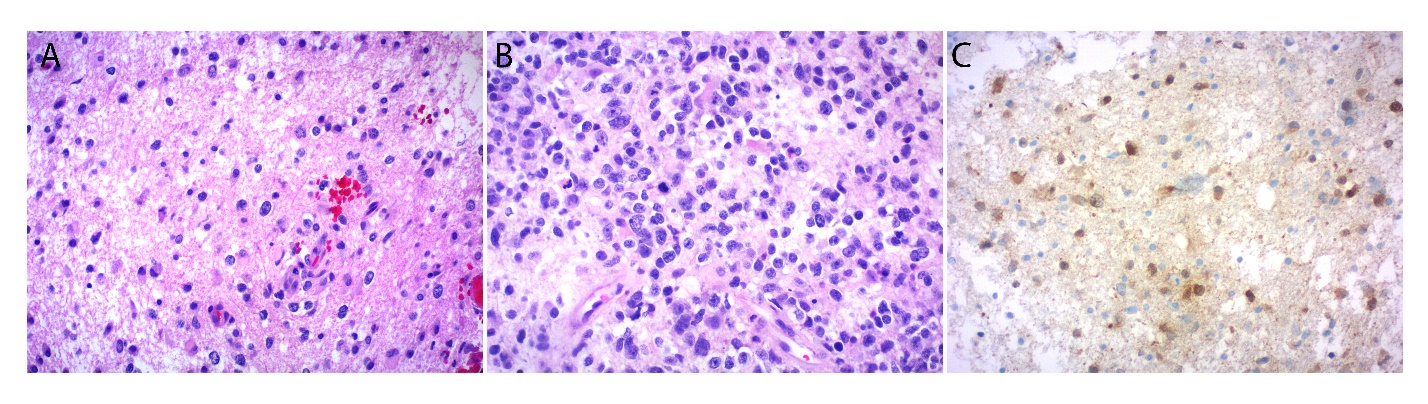
**

Separate and adjacent areas of lower grade (WHO grades II to III) infiltrating glioma (A, 200X; B, 200X). These areas were positive for IDH1 (R132H) mutant protein.

**Supplemental Figure 2**

**
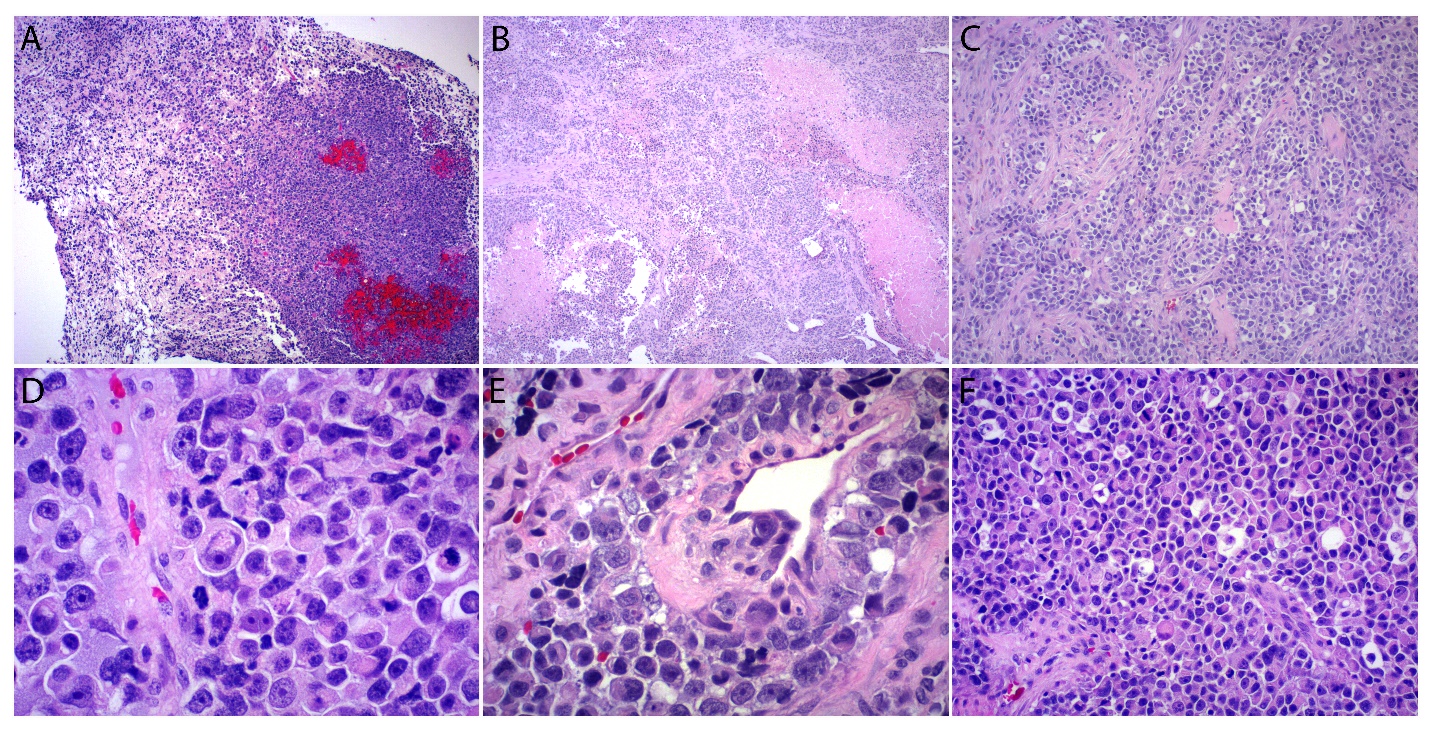
**

The tumor was largely composed of patternless sheets of cells (A, 100X; B, 40X; C, 200X) with rounded cytoplasmic contours, variable amounts of eosinophilic cytoplasm with minimal to no stellate cellular processes (D, 400X; E, 400X; F, 200X), large nuclei, and variably prominent nucleoli (D). Necrosis was predominantly zonal and frequently associated with sclerosed or thrombosed blood vessels (B, 40X). Rare foci suspicious for vascular invasion were also noted (E, 400X). Rare fragments showed adjacent areas of lower grade, infiltrating glioma (A).

**Supplemental Figure 3
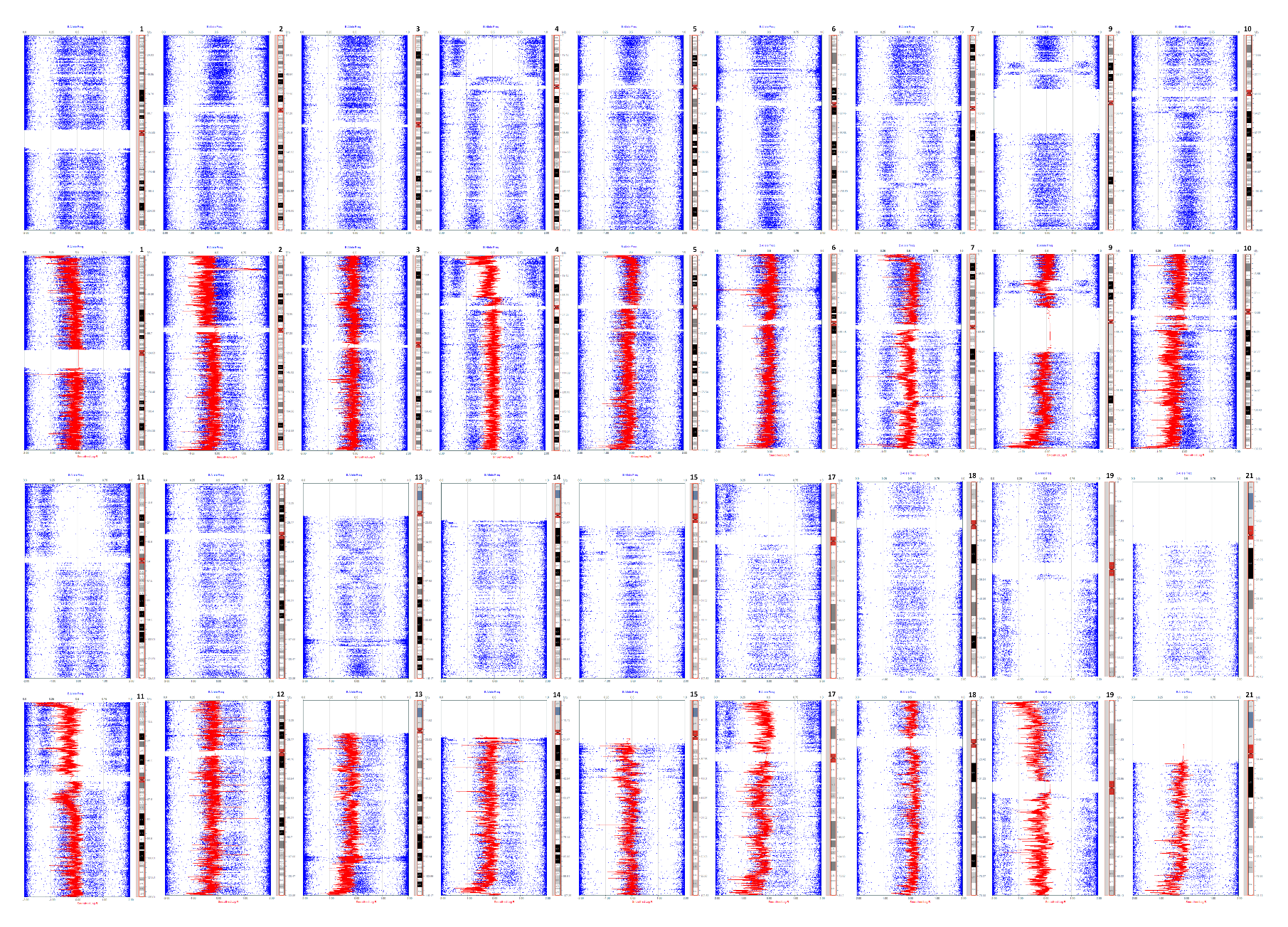
**

**Supplemental Table**
